# Sleep phenotypes and mental disorders: analysis of causality with two-sample Mendelian randomization

**DOI:** 10.1192/j.eurpsy.2025.2335

**Published:** 2025-08-26

**Authors:** M. Marchi, D. Uberti, E. Schiatti, K. Rachedi, G. M. Galeazzi, S. Ferrari

**Affiliations:** 1 Department of Biomedical, Metabolic and Neural Sciences, University of Modena and Reggio Emilia, Modena, Italy

## Abstract

**Introduction:**

Sleep health is s an essential aspect of a healthy lifestyle, and sleep problems are prevalent among individuals with mental disorders. However, this relationship appears complex to explore with classic observational studies, due to bidirectional effects and residual confounding. Additionally, the different measures of sleep quality and the diversity of mental disorders make comprehensive assessment challenging.

**Objectives:**

In the current study, we aimed at investigating the causal relationship between six sleep factors, derived from twelve indicators, and thirteen mental disorders. Specifically, we examined the impact on Alzheimer disease, attention-deficit and hyperactivity disorder (ADHD), anorexia nervosa, anxiety disorder, autism spectrum disorder, alcohol use disorder, bipolar disorder (BD), cannabis use disorder (CUD), major depressive disorder, obsessive-compulsive disorder, post-traumatic stress disorder, suicide attempt, and schizophrenia (SZ).

**Methods:**

Using Genomic Structural Equation Modeling, we estimated genome-wide associations for six sleep factors in the UK Biobank. Next, we examined bidirectional causal relationships with mental disorders in the Psychiatric Genomics Consortium, using Two-sample Mendelian Randomization (MR). Results are presented as inverse-variance weighted betas (B) with 95% confidence intervals (95%CI), representing log-odds for sleep-to-mental disorder causality (forward MR) and linear regression coefficients for mental disorder-to-sleep causality (backward MR).

**Results:**

Our investigation confirmed previous evidence of a six-factor model of sleep, comprising alertness (AF), circadian preference (CPF), efficiency, duration (DF), regularity, and insomnia (IF). MR analyses showed bidirectional causal relationship between IF and ADHD (B:0.747[95%CI:0.392;1.10] and B:0.029[95%CI:0.020;0.040] for forward and backward, respectively). Unidirectional causal effects were found for BD on AF (B:-0.113[95%CI:-0.153;-0.072]), SZ on AF (B:-0.057[95%CI:-0.077;-0.037]), BD on CPF (B:-0.066[95%CI:-0.104;-0.027]), CPF on ADHD (B:-0.074[95%CI:-0.113;-0.035]), BD on DF (B:0.038[95%CI:0.026;0.050]), SZ on DF (B:0.022[95%CI:0.016;0.029]), IF on CUD (B:0.764[95%CI:0.130;1.40]), and IF on SZ (B:-0.504[95%CI:-0.802;-0.206]).

**Conclusions:**

This study provide evidence that mental disorders negatively affect sleep quality rather than vice versa. These findings highlight the need to improve detection of sleep problems in mental health care settings and support efforts to identify intervention targets to improve sleep health among individuals with mental disorders.

**Disclosure of Interest:**

None Declared

